# Comparison of C-MAC D-blade videolaryngoscope and McCoy laryngoscope efficacy for nasotracheal intubation in simulated cervical spinal injury: a prospective randomized comparative study

**DOI:** 10.1186/s12871-020-01021-x

**Published:** 2020-05-14

**Authors:** Kwon Hui Seo, Kyung Mi Kim, Hyunji John, Joo Hyun Jun, Minsoo Han, Soyoun Kim

**Affiliations:** 1 Department of anesthesiology and pain medicine, Hallym University Sacred Heart Hospital, Hallym University School of Medicine, 22, Gwanpyeong-ro 170 beon-gil, Dong-gu, Anyang-si, Gyeonggi-do 14068 Republic of Korea; 2 Clinical assistant professor, Department of anesthesiology and pain medicine, Asan Medical Center, University of Ulsan College of Medicine, 88, Olympic-ro 43-gil, Songpa-gu, Seoul 05505 Republic of Korea; 3 Department of anesthesiology and pain medicine, Kangnam Sacred Heart Hospital, Hallym University School of Medicine, 12, Siheung-daero 187-gil, Yeongdeungpo-gu, Seoul, 07441 Republic of Korea

**Keywords:** Intubation, Intratracheal, Videolaryngoscope, Laryngoscopes, Cervical spine, Immobilization

## Abstract

**Background:**

Immobilization with cervical spine worsens endotracheal intubation condition. Though various intubation devices have been demonstrated to perform well in oral endotracheal intubation, limited information is available concerning nasotracheal intubation (NTI) in patients with cervical spine immobilization. The present study compared the performance of the C-MAC D-Blade videolaryngoscope with the McCoy laryngoscope for NTI in patients with simulated cervical spine injuries.

**Methods:**

This was a prospective, randomized, controlled, study done in a tertiary hospital. Ninety-five patients requiring NTI were included in data analysis: McCoy group (group M, *n* = 47) or C-MAC D-Blade videolaryngoscope group (group C, *n* = 48). A Philadelphia neck collar was applied before anesthetic induction to immobilize the cervical spine. Single experienced anesthesiologist performed NTI. The primary outcome was duration of intubation divided by three steps: nose to oropharynx; oropharynx into glottic inlet; and glottic inlet to trachea. Secondary outcomes included glottic view as percentage of glottis opening (POGO) score and Cormack-Lehance (CL) grade, modified nasal intubation-difficulty scale (NIDS) rating, hemodynamic changes before and after intubation, and complications.

**Results:**

Total intubation duration was significantly shorter in group C (39.5 ± 11.4 s) compared to group M (48.1 ± 13.9 s). Group C required significantly less time for glottic visualization and endotracheal tube placement in the trachea. More patients in group C had CL grade I and higher POGO scores (*P* <  0.001, for both measures). No difficulty in NTI (modified NIDS = 0) was more in group C than group M. Hemodynamic changes and incidence of complications were comparable between groups.

**Conclusion:**

The C-MAC D-Blade videolaryngoscope is an effective tool for NTI in a simulated difficult airway, which improves glottic visualization and shortens intubation time relative to those with McCoy laryngoscope.

**Trial registration:**

Clinical Research Information Service of the Korea National Institute of Health, Identification number: KCT 0004535, Registered December 10, 2019, Retrospectively registered, http://cris.nih.go.kr.

## Introduction

Inappropriate airway management in patients with cervical spine injuries can lead to deleterious effects on neurologic injury. For this reason, international guidelines recommend keeping the cervical spine in a neutral position and avoid movement of the cervical spine during endotracheal intubation with a rigid neck collar or manual in-line stabilization [1, 2]. It is well documented that immobilization of the cervical spine in patients with known or suspected cervical spine injuries is associated with increased rates of failed intubation of the trachea, secondary to adverse impact on the laryngeal view during direct laryngoscopy [3, 4]. NTI using fiber-optic bronchoscopy is a useful technique in patients in whom direct laryngoscopy and orotracheal intubation are impeded, for example, those with cervical spine injury [5]. However, it can be used on a limited basis because it requires experienced clinicians and takes longer to conduct than other devices. Recognition of these limitation had led to introduction of the variety of endotracheal intubation devices including various videolaryngoscopes to secure the airway for NTI in patients with cervical spine instability [6–8].

The McCoy laryngoscope, which is designed with a hinged tip at the end of the laryngoscope blade to facilitate easy lifting the epiglottis, has been documented to be a useful tool for orotracheal intubation by improving Cormack and Lehane (CL) laryngoscopic view in anticipated difficult intubation with cervical spine injury compared to conventional laryngoscope [9, 10].

The C-MAC videolaryngoscope, has a built-in light source and a digital camera, which allows for visualization of the larynx and vocal cords through a monitor while performing endotracheal intubation [11]. In particular, the C-MAC D-Blade videolaryngoscope has a noticeable curvature of the distal end of the blade, which faces markedly upward [12]. As a result of the exaggerated curvature of the blade components, a view of the glottis is provided without alignment of the oral, pharyngeal, or tracheal axes. The C-MAC D-Blade videolaryngoscope with extra-curved blade requires less cervical spine movement than conventional laryngoscopy with a Macintosh laryngoscope [13], therefore it achieved high intubation success rate with less tissue trauma in patients and manikin with neck stabilization [14, 15].

These two devices have been used successfully for orotracheal intubation in various anticipated difficult airway scenarios [10, 16, 17], but literature provides scant evidence for validating the use of McCoy blade and/or C-Mac D-Blade during NTI in cases with cervical injury or in a simulated difficult airway in humans. Thus, in the present study we explored the clinical performance of airway management with the McCoy laryngoscope and the C-MAC D-Blade videolaryngoscope for NTI in a simulated difficult airway with cervical spine immobilization in patients undergoing elective surgery.

## Methods

### Study population and ethical approval

The present study’s protocol was approved by the Sacred Heart Hospital, Hallym University, Institutional Review Board (approval No. 2018–04–024-004) and was registered with the Clinical Research Information Service of the Korea National Institute of Health (CRIS, http://cris.nih.go.kr, identification number: KCT 0004535). Written informed consent was obtained from all patients prior to any study-related procedures. The present study adhered to CONSORT guidelines.

One hundred patients scheduled for elective surgery under general anesthesia with NTI were enrolled in this prospective, randomized, controlled, study done in a tertiary hospital. All patients were between 20 and 80 years old and had an American Society of Anesthesiologists physical status of I–III. Patients were excluded if they had an anticipated difficult endotracheal intubation (Mallampati score IV and thyromental distance of ≤6.0 cm); a tendency toward bleeding; a history of nasal deformity, obstructive sleep apnea, recurrent epistaxis, nasopharyngeal abnormality or surgery; severe obesity (body mass index [BMI] ≥ 35 kg/m^2^); cervical spine instability; or cervical myelopathy.

### Randomization

All subjects were randomly assigned to one of two groups in a 1:1 ratio using a computer-generated random numbers table (www.randomizer.org). The randomization scheme was conducted by a resident anesthesiologist who was not involved in the data analysis or the anesthetic management. NTI was conducted using the McCoy laryngoscope (Optima, Timesco Ltd., London, England) in the group M, while NTI was conducted using a C-MAC D-Blade videolaryngoscope (Karl Storz, Tuttligen, Germany) in the group C. All subjects were blinded to their group assignment.

### Study protocol

All patients were premedicated with 0.2 mg of intramuscular glycopyrrolate 30 min before the anesthesia induction. Prior to their arrival in the operating theater, an attending anesthesiologist conducted an airway examination which included an assessment of the thyromental distance, inter-incisor distance, neck circumstance, and modified Mallampati score. Upon arrival in the operating theater, basic monitoring including electrocardiography, noninvasive blood pressure measurement, and pulse oximetry were employed in all cases. Patients were placed in the supine position with their head on an 8-cm-high pillow and asked which nostril was easier to breathe through to determine patency. If both nostrils had same patency, NTI was first attempted in the right nostril.

A pre-formed double-curved nasotracheal tube (Shilley™ Nasal RAE Tracheal Tube Cuffed; Covidien, Mansfield, MA, USA) with an internal diameter of 7.0 mm for men and 6.5 mm for women was used for endotracheal intubation. The ETT was thermosoftened for at least 30 min before intubation by placement into a bottle of sterile isotonic saline in a warming cabinet (KRS-205; Karis, Gyeonggi-do, Korea) at 45 °C [18]. Immediately before NTI, the ETT was withdrawn from the saline bottle and lubricated with water-soluble jelly. Before NTI, a cotton swab soaked with Bosmin solution^Ⓡ^ (0.1% epinephrine, 50 mL/bottle, Jeil Pharmaceutical Co., Seoul, Korea) was applied to both nasal cavities to prevent nasal bleeding.

Five minutes after preoxygenation, anesthesia was induced with propofol (1.5–2.0 mg/kg) and remifentanil (0.1–0.2 μg/kg/min). After anesthetic administration, the pillow was removed and a properly sized Philadelphia neck collar (Philadelphia Cervical Collar, Philadelphia Cervical Collar Co., Thorofare, NH, USA) was placed according to each patient’s neck circumstance or height. Following assessment of the ability to ventilate, rocuronium (0.6 mg/kg) was administered intravenously to facilitate NTI, and the patients’ lungs were manually ventilated with 2–4% sevoflurane in 100% oxygen. After 3 min, NTI was performed using the assigned device by single experienced anesthesiologist, who conducted at least 100 successful intubations using the McCoy laryngoscope and 100 successful intubations using the C-MAC D-Blade videolaryngoscope.

The primary outcome variable was total intubation time defined as the time from insertion of the ETT past the selected nostril to removal of intubation devices from oral cavity. Secondary outcomes, including time required for insertion of the ETT in each of three intubation steps (from nose into oropharynx, from oropharynx into glottic inlet, and from glottic inlet to trachea), navigation grade of ETT in each intubation steps, modified nasal intubation difficulty score (NIDS), hemodynamic changes, and several complications related to NTI.

The NTI was subdivided into three steps according to ETT passage as follows: 1) nose to oropharynx, 2) oropharynx into the glottic inlet, and 3) glottic inlet to trachea. The time taken and grade of difficulty were assessed for each of these three steps. In the first step, difficulty of ETT passage was graded as follows: grade 1) easy to advance, grade 2) slight resistance confronted, or grade 3) the ETT was unable to be advanced into the chosen nasal cavity and was inserted through contralateral nasal cavity. In the second step, difficulty was graded as follows: grade 1) smooth ETT passage from the oropharynx to the glottic inlet or grade 2) the ETT tip failed to align with the glottic inlet, and a lifting force or/and external pressure was applied to the larynx (BURP; backward, upward, and rightward pressure maneuver) to expose the vocal cords and align the ETT into glottic inlet. In the third step, ETT passage was graded as follows: grade 1) smooth passage of ETT into the trachea, grade 2) slight ETT manipulation (rotation and slight pressure) or/and Magill forceps were necessary to facilitate ETT advancement into the trachea.

To score the intubating conditions after NTI completion, a modified NIDS was used (Table 1). The total NIDS was categorized as follows: no difficulty (score = 0), mild difficulty (score between 0 and 5), moderate difficulty (score between 6 and 11), or profound difficulty (score of 12 or more) [19].

**Table 1:** Modified nasal intubation-difficulty scale (NIDS)

Parameters	Score
N1: Intubation attempts	Each additional intubation attempt after the first one adds 1 point
N2: Operators to attempt intubation	Each additional operator required to attempt intubation adds 1 point
N3: Alternative intubation techniques or change head position	Each alternative intubation technique or change head position adds 1 point
N4: Glottic exposure	0 = good visualization of vocal cords with little manipulation 1 = tools manipulated in all directions to identify the vocal cords 2 = tools extensively manipulated in all directions to identify the vocal cords
N5: Lifting force required to expose the vocal cords	0 = lifting without assistance 1 = lifting required by assistant to improve view of the vocal cords
N6: Optimize glottis exposure with BURP (backward, upward and right ward pressure)	0 = none 1 = BURP applied
N7: Techniques to aid intubation	0 = none 1 = cuff inflation or Magill forceps

The following data were also recorded. Based on the observed laryngoscopic view, the CL classification score [20] and percentage of glottic opening (POGO) scale [21] were noted for each patient. The POGO represented the glottic opening along the linear span from the anterior commissure to the inter-arytenoid notch. A 100% POGO was a full view of the glottis from the anterior commissure to the inter-arytenoid notch while a POGO of 0 indicated that even the inter-arytenoid notch was unseen. Mean arterial pressure (MAP), heart rate (HR), and peripheral oxygen saturation (SpO_2_) were recorded just before NTI, and at 1 and 3 min after intubation. In addition, 3 min after intubation, a separate anesthesiologist assessed for oropharyngeal bleeding by inspecting the laryngoscope blade for blood and the patient for mucosal bleeding. In the postoperative recovery room, complications related to NTI including a sore throat and hoarseness were evaluated.

Failure to intubate was defined as an inability to intubate the patient’s trachea within 120 s, or within three intubation attempts, or if the patient’s SpO_2_ dropped below 95% during NTI. In that case, the Philadelphia neck collar was removed and NTI was performed in the traditional position using the desired device by another skilled anesthesiologist with > 15 years of clinical experience.

Anesthesia was maintained with air and oxygen (50,50) and sevoflurane 2.0–3.5 vol%. Fentanyl (0.5 μg/kg) was administered 15 min before the end of surgery for postoperative analgesia.

### Statistical analyses

All quantitative variables including age, BMI, thyromental distance, neck circumstance, inter-incisor distance and total intubation time, time taken in each three steps, visual analog scale (VAS) of facemask ventilation difficulty, POGO score, VAS of sore throat were analyzed using descriptive statistics and summarized as means ± SDs or median (interquartile range 25th–75th). All qualitative variables (e.g., gender, ASA class, grade of difficulty, etc.) were presented as frequencies and percentages. Quantitative parameter normality was tested with the Shapiro–Wilk test. These outcomes were then assessed by a Student’s *t* test or the Mann–Whitney *U* test for independent groups, as appropriate. Qualitative data were assessed via the Chi square or Fisher’s exact test. For repeated measures including MAP and HR, a repeated measured ANOVA with adjustment for multiple comparisons via the Bonferroni post-hoc correction was used. All statistical analyses were performed using SPSS software version 24.0 (SPSS Inc., Chicago, IL, USA) for Windows (Microsoft Corporation, Redmond, WA, USA). *P* values < 0.05 were considered statistically significant for all parameters.

### Sample size

A necessary sample size calculation was conducted based on the primary outcome (the time taken for NTI) using G power. A pilot study revealed a time taken for NTI in the McCoy group of 48 s. A 10-s difference in the time taken for NTI between the C-MAC and McCoy groups was considered clinically significant based on a previous study [22], and the effect size was computed as 0.80. Based on this, we determined that forty-two patients were required in each group to detect 10-s difference in the total intubation time with 95% power, a significance level of 5%, and two-sided testing. Given a drop-out rate of 10%, we determined that a total of 95 patients were required.

## Results

### Patient characteristics

**Fig. 1:**
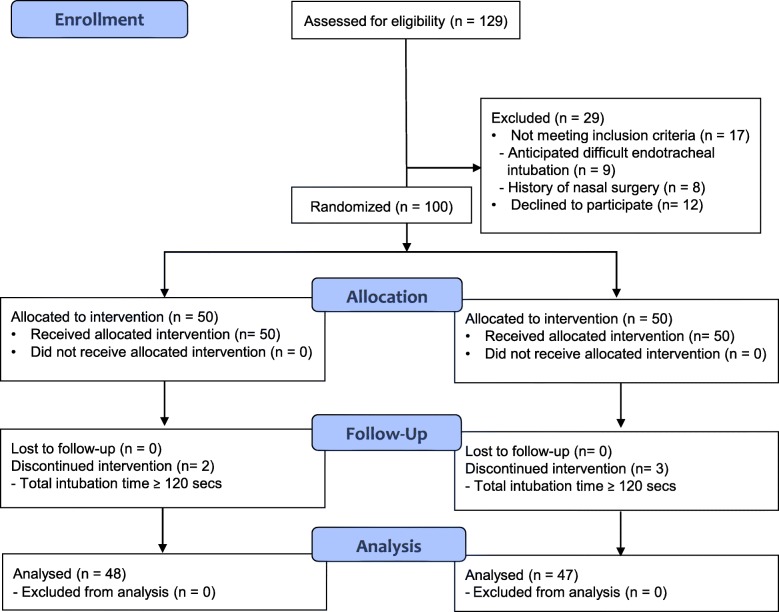
CONSORT diagram depicting the flow of participants

A total of 129 subjects were screened (Fig. 1). Seventeen subjects did not fulfill study inclusion criteria and 12 declined to participate. The 100 remaining eligible subjects were randomized (50 subjects in each group) and enrolled. Five subjects (two in group C and three in group M) were excluded because their total intubation time exceeded 120 s. Given this, 95 subjects completed the study according to protocol.

**Fig. 2:**
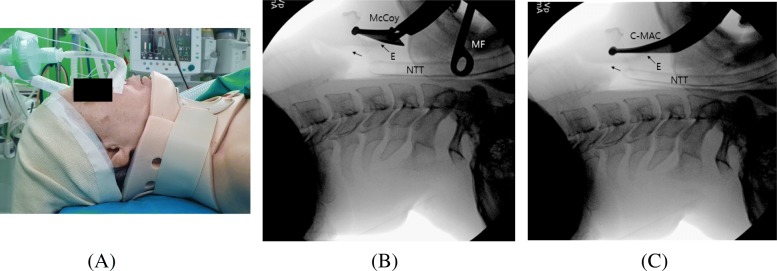
(**a**) Lateral picture of a patient with simulated cervical spine immobilization using the Philadelphia neck collar. (**b**) Lateral radiograph of a patient undergoing nasotracheal intubation with the McCoy laryngoscope. The McCoy laryngoscope significantly elevates the epiglottis; therefore, Magill forceps are frequently needed to navigate the nasotracheal tube into the glottic inlet. McCoy: McCoy laryngoscope, MF: Magill forceps, E: Epiglottis, NTT: nasotracheal tube, arrow without marking = glottic inlet. (**c**) Lateral radiograph of a patient undergoing nasotracheal intubation with C-MAC videolaryngoscope. C-MAC videolaryngoscope maintains the configuration of the airway in its original position, which allows for alignment of the nasal tube tip with the glottic inlet and smooth advancement of the NTT. C-MAC: C-MAC videolaryngoscope. The picture and radiographs were taken after obtaining informed consent

Patient demographic and preoperative data are represented in Table 2. There were no significant differences between groups in age, gender, ASA physical status, Mallampati class, thyromental distance, and neck circumference. The BMI for group M was significantly larger than that for group C (22.8 ± 3.5 kg/m^2^ vs. 24.4 ± 3.8 kg/m^2^, *P* = 0.033), but it was probably not practically/clinically significant. When the Philadelphia neck collar was placed, the inter-incisor distance decreased in both groups, though there was no significant difference between groups in inter-incisor distance after applying the Philadelphia neck collar. Pictures and lateral radiographs of a representative patient wearing the Philadelphia neck collar and then undergoing procedures with the C-MAC D-Blade videolaryngoscope or the McCoy laryngoscope were presented in Fig. 2.

**Table 2:** Demographic and preoperative data of patients

Patient characteristics	Group C (*n* = 48)	Group M (*n* = 47)
Age	47 (30.8–55.8)	36 (26.0–50.0)
Gender (M/F) (n)	29/19	27/20
BMI (kg/m^2^)	22.8 ± 3.5	24.4 ± 3.8
ASA class (n)		
I/II/III	10/33/5	17/26/4
Mallampati class (n)		
I/II/III/IV	21/18/5/4	16/19/10/2
Thyromental distance (cm)	8.0 (8.0–9.0)	8.0 (8.0–10.0)
Neck circumference (cm)	37.5 (34.0–39.8)	38.0 (36.0–40.0)
Inter-incisor distance (cm)		
Without collar	4.0 (4.0–5.0)	4.0 (4.0–5.0)
With collar	2.5 (2.0–3.0)	2.0 (2.0–3.0)

Values are expressed as mean ± SD, median (interquartile range 25th–75th) or number (%), *SD*Standard deviation

*BMI* Body mass index, *ASA* American society of anesthesiologists

### Intubation parameters

An ETT could be inserted into the nasopharynx of all patients in the present study. There was no significant difference in the grade of difficulty and required time between groups M and C during the first step of ETT passage (from nose to oropharynx) (Table 3).

**Table 3:** Intubation profiles compared between CMAC video-laryngoscope and McCoy laryngoscope according to each intubation step

Characteristics	Group C (*n* = 48)	Group M (*n* = 47)	*P* value
Nose to oropharynx			
Grade of difficulty			0.434
1	43 (89.6)	42 (89.4)	
2	2 (4.2)	4 (8.5)	
3	3 (6.3)	1 (2.1)	
Time (S)	9.6 ± 5.6	8.5 ± 2.6	0.563
Oropharynx to glottic inlet			
Grade of difficulty			< 0.001
1	37 (77.1)	12 (25.5)	
2	11 (22.9)	35 (74.5)	
Time (S)	13.0 ± 5.3	17.7 ± 7.7	0.004
Glottic inlet to trachea			
Grade of difficulty			0.002
1	18 (37.5)	5 (10.6)	
2	30 (62.5)	42 (89.4)	
Time (S)	16.8 ± 7.4	22.0 ± 11.4	0.027
Total intubation time (S)	39.5 ± 11.4	48.1 ± 13.9	0.004

Values are median ± SD or number of patients (%)

During the second step (navigating the ETT from the oropharynx to the glottic inlet), there was a significant difference in the navigation grade between the two groups (proportion of grade 1; 77.1% in group C vs. 25.5% in group M, *P* <  0.001). As for the time required in the second step, oropharynx to glottic opening, the McCoy laryngoscope took longer than C-MAC D-Blade videolaryngoscope (17.7 ± 7.7 s vs. 13 ± 5.3 s, *P* = 0.004). Furthermore, CL grade (I/II/III/IV) (25/16/7/0 vs. 4/19/16/8, *P* <  0.001) and POGO score (79.6 ± 20.6% vs. 50.6 ± 25.9%, *P* <  0.001) indicated significantly greater glottic visualization with the C-MAC D-Blade videolaryngoscope than with the McCoy laryngoscope (Table 4).

**Table 4:** Intubation profiles in the overall intubation period

Characteristics	Group C (*n* = 48)	Group M (*n* = 47)	*P* value
VAS of facemask ventilation difficulty	2.1 ± 1.6	2.6 ± 1.7	0.135
Cormack-Lehane grade (I/II/III/IV)	25/16/7/0	4/19/16/8	< 0.001
POGO scores (%)	79.6 ± 20.6	50.6 ± 25.9	< 0.001
NIDS			0.034
0 (no difficulty)	10 (20.8)	2 (4.3)	
1–5 (minor difficulty)	38 (79.2)	44 (93.6)	
6–11 (moderated difficulty)	0 (0)	1 (2.1)	
≥ 12 (profound difficulty)	0 (0)	0 (0)	
Use of Magill’s forceps (Yes/No)	27/21 (56.3/43.7)	40/7 (85.1/14.9)	0.002
Epistaxis (Yes/No)	8/40 (16.7/83.3)	5/42 (10.6/89.4)	0.504
Hoarseness (Yes/No)	0/48 (0/100)	1/46 (2.1/97.9)	0.310
VAS of sore throat	5.0 (4.0–6.0)	37.5 (34.0–39.8)	0.911

Values are median ± SD, median (interquartile range 25th–75th) or number (%)

*VAS* visual analog scale, *POGO* percentage of glottic opening, *NIDS* Nasal intubation difficulty scale

During the third step (inserting the ETT into the trachea from the glottic opening), there was a significant difference between the groups in terms of easiness of ETT advancement (proportion of grade 1; 37.5% in group C vs. 10.6% in group M, *P* = 0.042). Furthermore, the time required for insertion of the ETT into the trachea during this step was longer in group M than in group C (22.0 ± 11.4 s vs. 16.8 ± 7.4 s, *P* = 0.027).

**Fig. 3:**
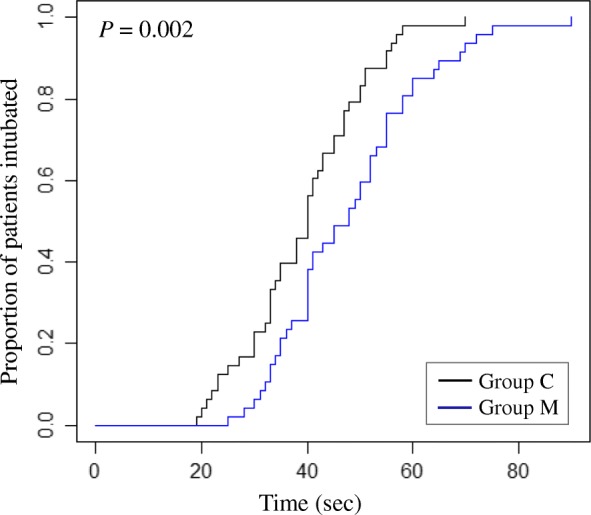
Kaplan-Meier plot demonstrating the proportion of patients intubated vs time. Pooled log-rank test. *P* = 0.002

The total time taken for complete NTI (the sum of the three intubation steps) was 9 s longer in group M than in group C (48.1 ± 13.9 s vs. 39.5 ± 11.4 s, *P* = 0.004, Table 3 and Fig. 3).

Patients who showed no difficulty in NTI (modified NIDS score = 0) were significantly more in patients with group C than group M (20.8% vs. 4.3%, *P* = 0.034). Also, Magill forceps were used approximately 29% more in group M than in group C (Table 4).

Hemodynamic parameters (MAP and HR) at 1 and 3 min after NTI were significantly increased in both groups when compared to pre-NTI values (*P* < 0.001, the two time points in MAP and HR of both groups). No intergroup differences were observed in MAP or HR (*P* = 0.257 and *P =* 0.632*,* respectively). The postoperative complications including epistaxis, sore throat, and hoarseness were comparable between groups (Table 4).

There was no significant difference between groups in incidence of failed intubation due to an inability to intubate the patient’s trachea within 120 s (4.0% (2/50) in group M vs. 6.0% (3/50) in group C, *P* = 1.0). There was no patient who was showed SpO_2_ < 95% during and after intubation. We compared SpO_2_ of two groups in just after intubation, there was no significant difference in SpO_2_ just after NTI (99% (99–100%) in group M vs. 100% (99–100%) in group C, *P* = 0.392).

## Discussion

In the present study, we compared the clinical performance of the C-MAC D-Blade videolaryngoscope and the McCoy laryngoscope for NTI in simulated cases of difficult airways with cervical spine immobilization. We found that the C-MAC D-Blade videolaryngoscope had significant benefits for intubation time and difficulty compared to the McCoy laryngoscopy during NTI. The C-MAC D-Blade videolaryngoscope provided for better glottic visualization and it took less time to advance the nasotracheal tube from the oropharynx to the glottic inlet with this device. Moreover, additional supporting maneuvers were required less in the C-MAC group compared to the McCoy group. Therefore, the C-MAC D-Blade videolaryngoscope was associated with a significantly lower modified NIDS score, indicating less difficulty during NTI.

While use of rigid collars in patients with suspected cervical spine injury effectively decreases cervical spinal movement and prevents the devastating neurological sequelae associated with laryngoscopy [23], it also worsens intubation conditions by impeding the appropriate alignment of the oropharyngeal-laryngeal axis and limiting the mouth opening [1, 3, 4]. Increased intubation times are related to these poor intubation conditions and are among the factors which influence hypoxia. Consequently, methods and devices that decrease intubation time can help to resolve the intubation problems associated with cervical spine immobilization.

The present study demonstrated that use of the C-MAC D-Blade videolaryngoscope was associated with a significantly greater frequency of CL grade I visualizations and higher POGO scores than the McCoy laryngoscope in a simulated difficult airway with cervical spine immobilization. These results agree with prior trials that suggested that the C-MAC D-Blade videolaryngoscope offers better glottic visualization in terms of a higher rate of CL grade I ratings compared with the McCoy laryngoscope during oropharyngeal intubation in patients who had limited neck mobility with a cervical collar [24]. These benefits on intubating condition may be explained by the pronounced curvature of the C-MAC D-Blade videolaryngoscope, which does not require alignment of oral, pharyngeal, and tracheal axes and its utility in cases of anterior placed larynx by providing an extended view of the vertical plane of the glottic areas [25, 26]. The result of the present study would be applied to patients with head and neck trauma, cervical spine disorders and difficult intubation anticipated.

The most time-consuming step of NTI is navigating the ETT, unlike orotracheal intubation in which the most time is taken to expose the glottis. The total time taken for complete NTI increases when additional supporting maneuvers, such as head position changes, application of BURP, rotation of the ETT, and use of Magill forceps, are required [27]. Numerous studies have shown that the use of a videolaryngoscope diminishes the necessity of these additional maneuvers, which may result in shorter NTI times as compared to the use of a direct laryngoscope. Tseung et al. [28]. reported that use of the GlideScope or Pentax AWS leads to reduced application of BURP, and Hazarika et al. [29]. reported that use of the C-MAC D-Blade videolaryngoscope resulted in less need for additional maneuvers such as tube rotation, cuff inflation, and the use of Magill forceps than use of Macintosh direct laryngoscopy. These findings agree with the results of the present study, which revealed that the use of additional maneuvers, including BURP and Magill forceps, occurred less in the C-MAC group than in the McCoy group. This significant difference in the necessity of additional maneuvers between the two groups likely contributed to the significant difference in intubation time in the present study.

The frequency of Magill forceps use was considerably higher in the McCoy group than in the C-MAC group (85.1% vs. 56.3%). The higher rate of Magill forceps use in the McCoy group may be attributable to its properties as a direct laryngoscope, which requires elevation of the laryngoscope blade, moves the larynx anteriorly and lengthens the distance between the glottic orifice and the posterior pharyngeal wall (Fig. 2B). These changes in the airway axis often require additional support maneuvers, such as lifting the head and Magill forceps to negotiate the nasal ETT into the glottic orifice [30]. On the contrary, the C-MAC D-Blade videolaryngoscope, which offers a non-line-of-sight view, often preserves the airway in its original configuration (Fig. 2C). Numerous trials have suggested that several intubating devices that provide non-line-of-sight views show a significantly improved conditions for NTI than conventional direct laryngoscopes. This is because these devices help to avoid deviation of the larynx from its original position and allow for easy placement of the ETT tip through the glottic inlet [29, 31, 32].

The hemodynamic response to endotracheal intubation is generally thought to result from irritation of the oropharyngeal tissue from laryngeal stimulation. Videolaryngoscopy requires less force on the base of the tongue than direct laryngoscopy to achieve a good glottic view. Thus, videolaryngoscopy is less likely to incite a pressor-response and causes laryngeal tissue injury [33]. The McCoy laryngoscope also has been reported to result in less hemodynamic change compared to the Macintosh laryngoscope [34, 35]. These two devices apply less lifting force than the Macintosh laryngoscope in endotracheal intubation. Although we did not compare the two devices to the Macintosh laryngoscope here, hemodynamic responses during NTI or peri-intubation periods were comparable between the two devices used in the present study.

There were several limitations the present study. First, the operator and the investigator recording laryngoscopic visualization and difficulty of intubation could not be blinded to the intubation device used. In addition, the single anesthesiologist conducted NTI. Therefore, observer bias may have impacted the result of the present study. If we had enrolled greater number of patients and several anesthesiologists had conducted NTI, the results could have been more objectively assessed. Secondly, the CL grading system has not been validated for evaluating the risk of difficult or failed intubation via videolaryngoscopy. Unfortunately, no other proper evaluation systems have been developed for this purpose. Thirdly, an experienced anesthesiologist conducted NTI, the effect of familiarity with the intubation device on the results cannot be excluded. Also, applying the results of the present study to beginners may be different in terms of time taken intubation and complications. Finally, BMI was significantly lower in the C-MAC group than in the McCoy group. It is conceivable that the lower BMI values of patients in the C-MAC group may have shortened the time required for NTI. However, the mean BMI values of both groups were less than 25 kg/m^2^ (22.2 ± 3.2 kg / m^2^ in the C-MAC group, 24.7 ± 3.9 kg / m^2^ in the McCoy group). Therefore, given that neither group had clinically significant mean BMI values, group-wise BMI differences may not have significantly affected the present study’s results.

In conclusion, C-MAC D-Blade videolaryngoscope-aided NTI is superior to that with McCoy laryngoscopy in terms of glottic visualization, ease of intubation, intubation time, and modified NIDS in patients with simulated cervical spinal injuries. Therefore, the C-MAC D-Blade videolaryngoscope is an effective tool for difficult airway management in patients requiring NTI with cervical spinal injuries. Further studies are needed to validate the performance of the C-MAC D-Blade videolaryngoscope in other clinical arenas.

## Data Availability

The data and materials are available from the corresponding author on reasonable request.

## Abbreviations

NTI: nasotracheal intubation
POGO: percentage of glottis opening
CL: Cormack-Lehance
NIDS: nasal intubation difficulty score
ETT: endotracheal tube
BMI: body mass index
BURP: backward, upward, and rightward pressure maneuver
MAP: mean arterial pressure
HR: heart rate
SpO_2_: peripheral oxygen saturation
VAS: visual analog scale
